# Supplementary material for: Dynalign II: common secondary structure prediction for RNA homologs with domain insertions
Source: Nucleic Acids Res. 2014 Nov 21;42(22):13939–48. doi: 10.1093/nar/gku1172 (PMC4267632; doi:10.1093/nar/gku1172)
Supplement: SUPPLEMENTARY DATA [file supp_gku1172_nar-02021-z-2014-File012.zip › manual/javadoc/RNAstructure_java_utilities/ScrollerPane.html]

ScrollerPane


JavaScript is disabled on your browser.


- Overview
- Package
- Class
- Tree
- Index
- Help

- Prev Class
- Next Class

- Frames
- No Frames

- All Classes

- Summary:
- Nested |
- Field |
- Constr |
- Method

- Detail:
- Field |
- Constr |
- Method


RNAstructure\_java\_utilities

## Class ScrollerPane

- java.lang.Object
- - java.awt.Component
  - - java.awt.Container
    - - javax.swing.JComponent
      - - javax.swing.JScrollPane
        - - RNAstructure\_java\_utilities.ScrollerPane

- All Implemented Interfaces:
  :   ImageObserver, MenuContainer, Serializable, Accessible, ScrollPaneConstants

  ---

    

  ```
  public class ScrollerPane
  extends JScrollPane
  ```

  A class that creates a scroll pane that can be controlled via the keyboard.

  See Also:
  :   Serialized Form

- - ### Nested Class Summary

    - ### Nested classes/interfaces inherited from class javax.swing.JScrollPane

      `JScrollPane.AccessibleJScrollPane, JScrollPane.ScrollBar`
    - ### Nested classes/interfaces inherited from class javax.swing.JComponent

      `JComponent.AccessibleJComponent`
    - ### Nested classes/interfaces inherited from class java.awt.Container

      `Container.AccessibleAWTContainer`
    - ### Nested classes/interfaces inherited from class java.awt.Component

      `Component.AccessibleAWTComponent, Component.BaselineResizeBehavior, Component.BltBufferStrategy, Component.FlipBufferStrategy`
  - ### Field Summary

    Fields

    | Modifier and Type | Field and Description |
    | --- | --- |
    | `static int` | `ALWAYS` A generic constant symbolizing that a scroll bar must always be used. |
    | `static int` | `NEVER` A generic constant symbolizing that a scroll bar must never be used. |
    | `private static long` | `serialVersionUID` |

    - ### Fields inherited from class javax.swing.JScrollPane

      `columnHeader, horizontalScrollBar, horizontalScrollBarPolicy, lowerLeft, lowerRight, rowHeader, upperLeft, upperRight, verticalScrollBar, verticalScrollBarPolicy, viewport`
    - ### Fields inherited from class javax.swing.JComponent

      `accessibleContext, listenerList, TOOL_TIP_TEXT_KEY, ui, UNDEFINED_CONDITION, WHEN_ANCESTOR_OF_FOCUSED_COMPONENT, WHEN_FOCUSED, WHEN_IN_FOCUSED_WINDOW`
    - ### Fields inherited from class java.awt.Component

      `BOTTOM_ALIGNMENT, CENTER_ALIGNMENT, LEFT_ALIGNMENT, RIGHT_ALIGNMENT, TOP_ALIGNMENT`
    - ### Fields inherited from interface javax.swing.ScrollPaneConstants

      `COLUMN_HEADER, HORIZONTAL_SCROLLBAR, HORIZONTAL_SCROLLBAR_ALWAYS, HORIZONTAL_SCROLLBAR_AS_NEEDED, HORIZONTAL_SCROLLBAR_NEVER, HORIZONTAL_SCROLLBAR_POLICY, LOWER_LEADING_CORNER, LOWER_LEFT_CORNER, LOWER_RIGHT_CORNER, LOWER_TRAILING_CORNER, ROW_HEADER, UPPER_LEADING_CORNER, UPPER_LEFT_CORNER, UPPER_RIGHT_CORNER, UPPER_TRAILING_CORNER, VERTICAL_SCROLLBAR, VERTICAL_SCROLLBAR_ALWAYS, VERTICAL_SCROLLBAR_AS_NEEDED, VERTICAL_SCROLLBAR_NEVER, VERTICAL_SCROLLBAR_POLICY, VIEWPORT`
    - ### Fields inherited from interface java.awt.image.ImageObserver

      `ABORT, ALLBITS, ERROR, FRAMEBITS, HEIGHT, PROPERTIES, SOMEBITS, WIDTH`
  - ### Constructor Summary

    Constructors

    Constructor and Description

    `ScrollerPane(Component comp,
    int width,
    int height)`

    Constructor.
  - ### Method Summary

    Methods

    | Modifier and Type | Method and Description |
    | --- | --- |
    | `void` | `setBarPolicies(int horizontal, int vertical)` Set the scroll bar policies for this scroll pane. |
    | `void` | `setSizes(int width, int height)` Set the size of the scroller pane by setting preferred, minimum, and maximum sizes all at once. |

    - ### Methods inherited from class javax.swing.JScrollPane

      `createHorizontalScrollBar, createVerticalScrollBar, createViewport, getAccessibleContext, getColumnHeader, getCorner, getHorizontalScrollBar, getHorizontalScrollBarPolicy, getRowHeader, getUI, getUIClassID, getVerticalScrollBar, getVerticalScrollBarPolicy, getViewport, getViewportBorder, getViewportBorderBounds, isValidateRoot, isWheelScrollingEnabled, paramString, setColumnHeader, setColumnHeaderView, setComponentOrientation, setCorner, setHorizontalScrollBar, setHorizontalScrollBarPolicy, setLayout, setRowHeader, setRowHeaderView, setUI, setVerticalScrollBar, setVerticalScrollBarPolicy, setViewport, setViewportBorder, setViewportView, setWheelScrollingEnabled, updateUI`
    - ### Methods inherited from class javax.swing.JComponent

      `addAncestorListener, addNotify, addVetoableChangeListener, computeVisibleRect, contains, createToolTip, disable, enable, firePropertyChange, firePropertyChange, firePropertyChange, fireVetoableChange, getActionForKeyStroke, getActionMap, getAlignmentX, getAlignmentY, getAncestorListeners, getAutoscrolls, getBaseline, getBaselineResizeBehavior, getBorder, getBounds, getClientProperty, getComponentGraphics, getComponentPopupMenu, getConditionForKeyStroke, getDebugGraphicsOptions, getDefaultLocale, getFontMetrics, getGraphics, getHeight, getInheritsPopupMenu, getInputMap, getInputMap, getInputVerifier, getInsets, getInsets, getListeners, getLocation, getMaximumSize, getMinimumSize, getNextFocusableComponent, getPopupLocation, getPreferredSize, getRegisteredKeyStrokes, getRootPane, getSize, getToolTipLocation, getToolTipText, getToolTipText, getTopLevelAncestor, getTransferHandler, getVerifyInputWhenFocusTarget, getVetoableChangeListeners, getVisibleRect, getWidth, getX, getY, grabFocus, isDoubleBuffered, isLightweightComponent, isManagingFocus, isOpaque, isOptimizedDrawingEnabled, isPaintingForPrint, isPaintingOrigin, isPaintingTile, isRequestFocusEnabled, paint, paintBorder, paintChildren, paintComponent, paintImmediately, paintImmediately, print, printAll, printBorder, printChildren, printComponent, processComponentKeyEvent, processKeyBinding, processKeyEvent, processMouseEvent, processMouseMotionEvent, putClientProperty, registerKeyboardAction, registerKeyboardAction, removeAncestorListener, removeNotify, removeVetoableChangeListener, repaint, repaint, requestDefaultFocus, requestFocus, requestFocus, requestFocusInWindow, requestFocusInWindow, resetKeyboardActions, reshape, revalidate, scrollRectToVisible, setActionMap, setAlignmentX, setAlignmentY, setAutoscrolls, setBackground, setBorder, setComponentPopupMenu, setDebugGraphicsOptions, setDefaultLocale, setDoubleBuffered, setEnabled, setFocusTraversalKeys, setFont, setForeground, setInheritsPopupMenu, setInputMap, setInputVerifier, setMaximumSize, setMinimumSize, setNextFocusableComponent, setOpaque, setPreferredSize, setRequestFocusEnabled, setToolTipText, setTransferHandler, setUI, setVerifyInputWhenFocusTarget, setVisible, unregisterKeyboardAction, update`
    - ### Methods inherited from class java.awt.Container

      `add, add, add, add, add, addContainerListener, addImpl, addPropertyChangeListener, addPropertyChangeListener, applyComponentOrientation, areFocusTraversalKeysSet, countComponents, deliverEvent, doLayout, findComponentAt, findComponentAt, getComponent, getComponentAt, getComponentAt, getComponentCount, getComponents, getComponentZOrder, getContainerListeners, getFocusTraversalKeys, getFocusTraversalPolicy, getLayout, getMousePosition, insets, invalidate, isAncestorOf, isFocusCycleRoot, isFocusCycleRoot, isFocusTraversalPolicyProvider, isFocusTraversalPolicySet, layout, list, list, locate, minimumSize, paintComponents, preferredSize, printComponents, processContainerEvent, processEvent, remove, remove, removeAll, removeContainerListener, setComponentZOrder, setFocusCycleRoot, setFocusTraversalPolicy, setFocusTraversalPolicyProvider, transferFocusDownCycle, validate, validateTree`
    - ### Methods inherited from class java.awt.Component

      `action, add, addComponentListener, addFocusListener, addHierarchyBoundsListener, addHierarchyListener, addInputMethodListener, addKeyListener, addMouseListener, addMouseMotionListener, addMouseWheelListener, bounds, checkImage, checkImage, coalesceEvents, contains, createImage, createImage, createVolatileImage, createVolatileImage, disableEvents, dispatchEvent, enable, enableEvents, enableInputMethods, firePropertyChange, firePropertyChange, firePropertyChange, firePropertyChange, firePropertyChange, firePropertyChange, getBackground, getBounds, getColorModel, getComponentListeners, getComponentOrientation, getCursor, getDropTarget, getFocusCycleRootAncestor, getFocusListeners, getFocusTraversalKeysEnabled, getFont, getForeground, getGraphicsConfiguration, getHierarchyBoundsListeners, getHierarchyListeners, getIgnoreRepaint, getInputContext, getInputMethodListeners, getInputMethodRequests, getKeyListeners, getLocale, getLocation, getLocationOnScreen, getMouseListeners, getMouseMotionListeners, getMousePosition, getMouseWheelListeners, getName, getParent, getPeer, getPropertyChangeListeners, getPropertyChangeListeners, getSize, getToolkit, getTreeLock, gotFocus, handleEvent, hasFocus, hide, imageUpdate, inside, isBackgroundSet, isCursorSet, isDisplayable, isEnabled, isFocusable, isFocusOwner, isFocusTraversable, isFontSet, isForegroundSet, isLightweight, isMaximumSizeSet, isMinimumSizeSet, isPreferredSizeSet, isShowing, isValid, isVisible, keyDown, keyUp, list, list, list, location, lostFocus, mouseDown, mouseDrag, mouseEnter, mouseExit, mouseMove, mouseUp, move, nextFocus, paintAll, postEvent, prepareImage, prepareImage, processComponentEvent, processFocusEvent, processHierarchyBoundsEvent, processHierarchyEvent, processInputMethodEvent, processMouseWheelEvent, remove, removeComponentListener, removeFocusListener, removeHierarchyBoundsListener, removeHierarchyListener, removeInputMethodListener, removeKeyListener, removeMouseListener, removeMouseMotionListener, removeMouseWheelListener, removePropertyChangeListener, removePropertyChangeListener, repaint, repaint, repaint, resize, resize, setBounds, setBounds, setCursor, setDropTarget, setFocusable, setFocusTraversalKeysEnabled, setIgnoreRepaint, setLocale, setLocation, setLocation, setName, setSize, setSize, show, show, size, toString, transferFocus, transferFocusBackward, transferFocusUpCycle`
    - ### Methods inherited from class java.lang.Object

      `clone, equals, finalize, getClass, hashCode, notify, notifyAll, wait, wait, wait`

- - ### Field Detail


    - #### ALWAYS

      ```
      public static final int ALWAYS
      ```

      A generic constant symbolizing that a scroll bar must always be used.

      See Also:
      :   Constant Field Values


    - #### NEVER

      ```
      public static final int NEVER
      ```

      A generic constant symbolizing that a scroll bar must never be used.

      See Also:
      :   Constant Field Values


    - #### serialVersionUID

      ```
      private static final long serialVersionUID
      ```

      See Also:
      :   Constant Field Values
  - ### Constructor Detail


    - #### ScrollerPane

      ```
      public ScrollerPane(Component comp,
                  int width,
                  int height)
      ```

      Constructor.

      Parameters:
      :   `comp` - The component to put in the scroll pane.
      :   `width` - The width of the scroll pane.
      :   `height` - The height of the scroll pane.
  - ### Method Detail


    - #### setBarPolicies

      ```
      public void setBarPolicies(int horizontal,
                        int vertical)
      ```

      Set the scroll bar policies for this scroll pane.

      Parameters:
      :   `horizontal` - The horizontal policy.
      :   `vertical` - The vertical policy.


    - #### setSizes

      ```
      public void setSizes(int width,
                  int height)
      ```

      Set the size of the scroller pane by setting preferred, minimum, and
      maximum sizes all at once.

      Parameters:
      :   `width` - The width of the scroll pane.
      :   `height` - The height of the scroll pane.


- Overview
- Package
- Class
- Tree
- Index
- Help

- Prev Class
- Next Class

- Frames
- No Frames

- All Classes

- Summary:
- Nested |
- Field |
- Constr |
- Method

- Detail:
- Field |
- Constr |
- Method
